# Supplementary material for: Na+-H+ exchanger 1 determines atherosclerotic lesion acidification and promotes atherogenesis
Source: Nat Commun. 2019 Sep 4;10:3978. doi: 10.1038/s41467-019-11983-3 (PMC6726618; doi:10.1038/s41467-019-11983-3)
Supplement: Supplementary file 2 — Reporting Summary [file 41467_2019_11983_MOESM2_ESM.pdf]

nature research

Corresponding author(s): Guo-Ping Shi, Junli Guo, Jin-Ying Zhang

Last updated by author(s): Aug 14, 2019

Reporting Summary

Nature Research wishes to improve the reproducibility of the work that we publish. This form provides structure for consistency and transparency in reporting. For further information on Nature Research policies, see Authors & Referees and the Editorial Policy Checklist.

Statistics

For all statistical analyses, confirm that the following items are present in the figure legend, table legend, main text, or Methods section.

n/a	Confirmed	
☐	☒	The exact sample size (n) for each experimental group/condition, given as a discrete number and unit of measurement
☐	☒	A statement on whether measurements were taken from distinct samples or whether the same sample was measured repeatedly
☐	☒	The statistical test(s) used AND whether they are one- or two-sided<br>*Only common tests should be described solely by name; describe more complex techniques in the Methods section.*
☐	☒	A description of all covariates tested
☐	☒	A description of any assumptions or corrections, such as tests of normality and adjustment for multiple comparisons
☐	☒	A full description of the statistical parameters including central tendency (e.g. means) or other basic estimates (e.g. regression coefficient) AND variation (e.g. standard deviation) or associated estimates of uncertainty (e.g. confidence intervals)
☒	☐	For null hypothesis testing, the test statistic (e.g. F, t, r) with confidence intervals, effect sizes, degrees of freedom and P value noted<br>*Give P values as exact values whenever suitable.*
☒	☐	For Bayesian analysis, information on the choice of priors and Markov chain Monte Carlo settings
☒	☐	For hierarchical and complex designs, identification of the appropriate level for tests and full reporting of outcomes
☒	☐	Estimates of effect sizes (e.g. Cohen's d, Pearson's r), indicating how they were calculated

Our web collection on statistics for biologists contains articles on many of the points above.

Software and code

Policy information about availability of computer code

Data collection	*Provide a description of all commercial, open source and custom code used to collect the data in this study, specifying the version used OR state that no software was used.*
Data analysis	SPSS 20.0 and Prism 7 (GraphPad) software were used for statistical analyses.

For manuscripts utilizing custom algorithms or software that are central to the research but not yet described in published literature, software must be made available to editors/reviewers. We strongly encourage code deposition in a community repository (e.g. GitHub). See the Nature Research guidelines for submitting code & software for further information.

Data

Policy information about availability of data

All manuscripts must include a data availability statement. This statement should provide the following information, where applicable:

- Accession codes, unique identifiers, or web links for publicly available datasets
- A list of figures that have associated raw data
- A description of any restrictions on data availability

Data supporting the findings of this manuscript are available from the corresponding author upon reasonable request. The source data underlying Figures 2a-j, 3c/f/Ii, 4a-c, 5a/c, 6a/b, 7a/c/d/e are provided as a Source Data file.

Field-specific reporting

Please select the one below that is the best fit for your research. If you are not sure, read the appropriate sections before making your selection.

☒ Life sciences ☐ Behavioural & social sciences ☐ Ecological, evolutionary & environmental sciences

For a reference copy of the document with all sections, see nature.com/documents/nr-reporting-summary-flat.pdf

Life sciences study design

All studies must disclose on these points even when the disclosure is negative.

Sample size	Sample sizes were assessed using the power calculation at $p<0.05$, power >0.80.
Data exclusions	No data were excluded from the analyses.
Replication	Each independent experiment was replicated at least 3 times.
Randomization	Mice in this study were allocated based on the availability.
Blinding	The investigators were blinded to group allocation during data collection and analysis.

Reporting for specific materials, systems and methods

We require information from authors about some types of materials, experimental systems and methods used in many studies. Here, indicate whether each material, system or method listed is relevant to your study. If you are not sure if a list item applies to your research, read the appropriate section before selecting a response.

Materials & experimental systems

n/a	Involved in the study	
☐	☒	Antibodies
☐	☒	Eukaryotic cell lines
☒	☐	Palaeontology
☐	☒	Animals and other organisms
☐	☒	Human research participants
☒	☐	Clinical data

Methods

n/a	Involved in the study	
☒	☐	ChIP-seq
☒	☐	Flow cytometry
☒	☐	MRI-based neuroimaging

Antibodies

Antibodies used

mouse anti-human IgE Fc-UNLB, Cat# 9160-01, SouthernBiotech, Birmingham, AL
anti-human CD68, Cat# M0814, Dako, Carpinteria, CA
anti-mouse Mac-3, Cat# 553322, BD Biosciences, San Jose, CA
Anti-mouse CD4, Cat# 553043, BD Biosciences, San Jose, CA
Anti-mouse α-actin, Cat# F3777, Sigma-Aldrich, St. Louis, MO
goat polyclonal FcεR1α antibody (G-14), Cat# sc-33484, Santa Cruz Biotechnology, Dallas, TX
rabbit polyclonal NHE-1 antibody (H-160), Cat# sc-28758, Santa Cruz Biotechnology, Dallas, TX
rat anti-mouse/human Mac-2, Cat# CL8942LE, Cedarlane, Burlington, Ontario, Canada
Alexa Fluor 488, Cat# A-11006, Thermo Fisher Scientific, San Diego, CA
Alexa Fluor 555, Cat# A-21432, Thermo Fisher Scientific, San Diego, CA
Alexa Fluor 647, Cat# A-21244, Thermo Fisher Scientific, San Diego, CA
DAPI, Cat# R37606, Thermo Fisher Scientific, San Diego, CA
mouse IgE (SPE-7), Cat# D8406, Sigma-Aldrich, St. Louis, MO
mouse anti-Nhe1 antibody, Cat# MAB3140, Sigma-Aldrich, St. Louis, MO
IgG isotype control antibody, Cat# 026502, Thermo Fisher Scientific, San Diego, CA
hamster anti-mouse FcεR1 antibody, #14-5898-82, Thermo Fisher Scientific, San Diego, CA
horseradish peroxidase (HRP)-conjugated mouse anti-hamster antibody, Cat# sc-2789, Santa Cruz Biotechnology, Dallas, TX
mouse phosphorylated-AKT, Cat# 2965S, Cell Signaling Technology, Danvers, MA
total AKT, Cat# 2920S, Cell Signaling Technology, Danvers, MA
phosphorylated-PI3 kinase, Cat# 4228S, Cell Signaling Technology, Danvers, MA
total PI3 kinase, Cat# 4257S, Cell Signaling Technology, Danvers, MA
phosphorylated-mTOR, Cat# 5536T, Cell Signaling Technology, Danvers, MA
total mTOR, Cat# 2972S, Cell Signaling Technology, Danvers, MA
β-actin, Cat# 8457S, Cell Signaling Technology, Danvers, MA
rat anti-mouse IgE antibody, Cat# 93236, BioLegend, San Diego, CA

	rat IgG1 κ isotype, Cat# 92233, BioLegend, San Diego, CA
Validation	We did not validate these commercial antibodies.

Eukaryotic cell lines

Policy information about cell lines

Cell line source(s)	Cell culture experiments were restricted to the use of mouse primary macrophages from bone marrow differentiation.
Authentication	N/A
Mycoplasma contamination	Not tested.
Commonly misidentified lines (See ICLAC register)	N/A

Animals and other organisms

Policy information about studies involving animals; ARRIVE guidelines recommended for reporting animal research

Laboratory animals	Both Nhe1+/– (C57BL/6 background, Cat# 003012) and Apoe–/– mice (C57BL/6 background, Cat#002052) were purchased from the Jackson Laboratory (Bar Harbor, ME), and crossbred to generate Apoe–/–Nhe1+/– and Apoe–/–Nhe1+/+ littermate controls. We used 8-week old Apoe–/–Nhe1+/– and Apoe–/–Nhe1+/+ male mice. We fed mice an atherogenic diet (Cat# D12108c, Research Diets Inc., New Brunswick, NJ) for 3 months to develop atherosclerosis. We also used wild type (WT) mice (C57BL/6, purchased from Jackson Lab) as experimental controls. Apoe–/–Fcer1a–/– and Apoe–/–Fcer1a+/+ mice were reported in our earlier study (EMBO Mol Med. 2014;6:952-969).<br><br>Male 8-week-old Apoe–/– mice were subjected to 1,000 rad of total body irradiation, followed by reconstitution with 2x10^6 bone-marrow cells from 8-week-old male Apoe–/– Nhe1+/+, Apoe–/–Nhe1+/–, Apoe–/–Fcer1a–/–, and Apoe–/–Fcer1a+/+ mice via tail-vein injection. All recipient animals were allowed to recover for 4 weeks on a standard chow diet after bone-marrow transplantation. The bone marrow-reconstituted mice then consumed an atherogenic diet for 3 months to develop atherosclerosis.
Wild animals	N/A
Field-collected samples	N/A
Ethics oversight	All animal procedures conformed to the Guide for the Care and Use of Laboratory Animals published by the US National Institutes of Health and was approved by the Brigham and Women's Hospital Standing Committee on Animals (protocol #2016N000442).

Note that full information on the approval of the study protocol must also be provided in the manuscript.

Human research participants

Policy information about studies involving human research participants

Population characteristics	Discarded and non-coded human carotid atherosclerotic lesion segments.
Recruitment	N/A. No new recruitment involved. No informed consent from patients required.
Ethics oversight	Discarded and decoded human aortas were reused according to the protocol #2010P001930 pre-proved by the Human Investigation Review Committee at the Brigham and Women's Hospital, Boston, Mass. No patient informed consent was required.

Note that full information on the approval of the study protocol must also be provided in the manuscript.
